# Parental Beliefs and Attitudes Toward the Use of Baby Walkers in the Eastern Region of Saudi Arabia

**DOI:** 10.7759/cureus.48940

**Published:** 2023-11-17

**Authors:** Mohammed Alquraini, Rawabi AlWasaifer, Rawan Alturki, Hajer AlSaif, Abdullah Alhashim, Dalal Bubshait

**Affiliations:** 1 Paediatrics and Child Health, King Faisal University, Al-Ahsa, SAU; 2 Orthopaedics, King Faisal University, Al-Ahsa, SAU; 3 Orthopedics, College of Medicine, Imam Abdulrahman Bin Faisal University, Dammam, SAU

**Keywords:** perception, saudi arabia, parental survey, orthopedics, pediatrics

## Abstract

Background

Baby walkers (BWs) are popular among parents worldwide, despite safety concerns and developmental impact concerns, as they are influenced by cultural beliefs, social myths, and personal interests. This study aims to assess parental beliefs and attitudes toward the use of BWs in the Eastern region of Saudi Arabia.

Materials and methods

A descriptive cross-sectional study was conducted among 400 mothers in the Eastern region of Saudi Arabia. Data were collected through an online questionnaire, which included demographic information, BW usage, reasons for usage/non-usage, and awareness of the dangers and disadvantages of BWs. Descriptive statistics and chi-square tests were used for data analysis.

Results

Among the participants, 332 (83.0%) reported using BWs for their children. The majority of parents i.e. 237 parents (71.3%) used walkers for their child’s fun and 146 parents (43.9%) used them for 1-2 hours daily. Among the reasons for non-usage, concerns about affecting the child's walking and potential injuries were most common in 29 (42.6%) and 28 (41.1%) parents, respectively. Significant associations were found between mother's age, child's birth order, age of crawling, age of independent walking, and BW usage. Forty-eight children (14.4%) who used walkers experienced injuries, including falling downstairs 20 (41.6%) and flipping over on a flat surface 21 (43.7%).

Conclusion

This study highlights the prevalence of BW usage and the reasons behind parental decisions in the Eastern region of Saudi Arabia. While many parents use BWs to promote early walking and provide entertainment, concerns about safety and potential developmental impacts persist.

## Introduction

Baby walkers (BWs) are fun, entertaining items used for infants aged 4 to 12 months around the world [1]. It is noticeable that the use of BWs is popular among families from different communities around the world as this was apparent from many studies that were done in the last 30 years, which showed usage rates between 47% and 83% in infants younger than 15 months of age [2]. It is well understood that parental decisions play a significant role in daily practices with the child. Their choices and behaviors regarding their children appear to be influenced more markedly by the values and beliefs that underpin their understanding and action than by guidance from health professionals or scientific evidence [3]. So, many parents use BWs based on cultural beliefs, social myths, and personal interests [1]. The main reasons for parents to use a BW are as follows: providing enjoyment, facilitating child development, assisting the child to walk, home safety, keeping the child quiet, encouraging mobility, providing exercise, and others [1].

The sale of BWs is prohibited in Canada. The American Academy of Pediatrics (AAP) wishes for the same in the United States. This is because BWs are risky. According to a study published in the journal Pediatrics, over 230,000 children under the age of 15 months were treated in US emergency departments for injuries caused by walkers between 1990 and 2014 [4].

When we talk about the harms of BWs, the Harvard Child and Adolescent Health Journal stated that most injuries occur when children fall downstairs in a walker, usually injuring their head or neck. It is not only stairs that can be problematic. Children in walkers may get their fingers caught, pull things down on themselves, or grab potentially dangerous items (such as sharp objects or hot liquids) that would otherwise be out of reach. Children in walkers can fall out and get hurt, and they have drowned when they scooted into a pool or spa. Toys attached to a BW have also caused injuries [4].

Thinking about alternatives to walkers will most likely be an important part of parental education. Stationary walkers are popular among parents in the United States because they pose a lower risk of injury while performing many of the same functions as a mobile walker. Furthermore, the gaps in knowledge about walker-related injuries suggest that pediatricians and pediatric orthopedic doctors should be further educated [5].

In this study, we aimed to build upon the existing body of research by investigating the prevalence of BW usage among children in the Eastern province of Saudi Arabia. Our study addresses a critical knowledge gap in the region, given the scarcity of data on this subject. The aims of our research are threefold: firstly, to estimate the prevalence of BW usage; secondly, to uncover the parental motivations and factors contributing to the utilization of BW; and lastly, to assess the level of awareness among parents regarding the potential dangers and disadvantages associated with their use. The importance of this study lies in its potential to inform public health initiatives and guide educational efforts aimed at ensuring the safety and well-being of children in our region.

## Materials and methods

Study design, setting, and participants

This cross-sectional study was conducted in the Eastern region of Saudi Arabia, specifically encompassing six administrative regions: Al-Ahsa, Al-Qatif, Al-Dammam, Al-Khobar, Al-Dhahran, and Al-Jubail. The study was carried out over a period of three months spanning from July 20 to October 1, 2023. The primary objective of the study was to assess parents' beliefs concerning the use of BWs within the Eastern region of Saudi Arabia. The inclusion criteria include mothers residing in the Eastern region who had at least one child. Ethical approval was obtained from the ethical committee of King Faisal University located in Al-Ahsa (reference number: KFU-REC-2023-OCT-ETHICS1223). Participation was voluntary, and the questionnaire was distributed online while ensuring participants' confidentiality. The required sample size for the study was determined to be 385 participants, calculated using the formula n=Z2 pq/E2. In this formula, the margin of error (E) was set at 0.05. Additionally, a confidence level (Za/2) of 95% was chosen, corresponding to a value of 1.96. The expected proportion (p) of adults in the population was considered to be 0.5. 

Questionnaire development and data collection

The data for this study were obtained by administering an online questionnaire to participants, after obtaining their informed consent. The online questionnaire was developed following established guidelines for questionnaire design. A thorough literature review was conducted to identify relevant constructs and existing measurement instruments in the field. Based on this review, an initial draft of the questionnaire was created which included items addressing the key variables of interest. To establish face validity, the initial draft questionnaire was presented to a group of mothers who met the inclusion criteria for the study. These participants were asked to provide feedback on the questionnaire's clarity, relevance, and overall design. Their input was invaluable in identifying any ambiguous or confusing items, and we made appropriate revisions based on their suggestions. This iterative process ensured that the questionnaire appeared meaningful and well-designed to the target population. Furthermore, to ensure content validity, the expertise of three independent researchers specializing in the field of Orthopedics was sought. These experts evaluated the questionnaire for its appropriateness, clarity, coverage, and relevance to the study objectives. They provided valuable insights and suggestions for improving the questionnaire's content validity. We incorporated their feedback by modifying and refining the questionnaire accordingly. Regarding reliability, the internal consistency of the questionnaire was assessed using Cronbach's alpha coefficient. A pilot study was conducted with a small sample of participants to calculate the coefficient, which yielded a value of 0.857, indicating a high level of internal consistency.

The questionnaire consists of three sections. The first section focuses on collecting personal and demographic information, such as age, nationality, educational level, place of residency, and number of children. The second section includes questions related to the child's motor milestones, specifically the age at which they began crawling and independently walking, as well as whether the parents utilized a BW. The third section of the questionnaire diverges into two distinct pathways based on the parents' use of a BW. For those who reported using a BW, questions were asked regarding the age at which the child started using the walker, reasons for using it, frequency of daily and weekly use, and any BW-related injuries. For parents who did not use a BW, questions were posed regarding their reasons for not using one and the source of information they relied upon. 

Statistical analysis 

The responses obtained from the questionnaire were summarized using descriptive statistics. Sociodemographic characteristics and other categorical variables were analyzed by calculating frequency distributions and percentages, which were then presented in tabular form. To examine the association between variables, the appropriate statistical test was chosen based on the nature of the research questions and the types of variables involved. The chi-square test was used where all the cells have a value greater than 5, and Fisher's exact test was employed for variables when at least one cell has a value less than 5, taking into consideration the potential impact of small sample sizes on the validity of the chi-square test. The choice between the chi-square test and Fisher's exact test ensured accurate analysis and appropriate interpretation of the results. All statistical analyses were performed using IBM SPSS Statistics for Windows, Version 29 (Released 2022; IBM Corp., Armonk, New York, United States). A significance level of p < 0.05 was used to determine statistical significance.

## Results

Our study includes 400 mothers assessing their use of BWs for their children. Table 1 shows the sociodemographic features of the participants. Regarding age distribution, 228 (57%) of participants were aged 21-30 years, and 163 (40.8%) of participants were residents of Al-Ahsa. Most mothers had a university education 308 (77%), with 167 (41.8%) employed. One hundred and fifty-two mothers (38%) had caregivers for their babies at home, and 329 (82.3%) of mothers had family support.

**Table 1:** Sociodemographic features of mothers assessing for baby walker use

		Frequency (n=400)	Percent
Age	< 20 Years	64	16.0
	21-30 Years	228	57.0
	31-40 Years	70	17.5
	41-50 Years	6	1.5
	51-60 Years	3	.8
	Missing Values	29	7.2
City of Residency	Dammam	108	27.0
	Dhahran	29	7.2
	Al-Ahssa	163	40.8
	Jubail	12	3.0
	Khobar	64	16.0
	Qatif	24	6.0
Mother Educational Level	< High School	20	5.0
	High School	72	18.0
	University	308	77.0
Mother Work	Yes	167	41.8
Any Caregiver at home	Yes	152	38.0
Family Support for Mother	Yes	329	82.3

Table 2 shows the features of babies assessing for BW use. Half of the babies (203) were females (50.7%). Notably, 288 (72.0%) of families had more than one child, with 262 (65.5%) being firstborns. A significant proportion of infants i.e. 306 infants (76.6%) crawled between 4 and 8 months, and most infants i.e. 223 infants (55.8%) walked independently between 12 and 15 months. 

**Table 2:** Features of children involved

		Frequency (n=400)	Percent
Child’s Gender	Male	197	49.3
	Female	203	50.7
No. of Children in Family	1	112	28.0
	> 1	288	72.0
Order of Child	First	262	65.5
	Middle	43	10.8
	Last	95	23.8
Age of Crawling of Child	Didn't Crawl Yet	13	3.3
	4-6 Months	109	27.3
	6-8 Months	197	49.3
	9-11 Months	69	17.3
	12 Months & Above	12	3.0
Age of Walking of Child Independently	Didn't Independently Walk Yet	31	7.8
	Before 12 Months	111	27.8
	12-15 Months	223	55.8
	15-18 Months	26	6.5
	After 18 Months	9	2.3

Table 3 shows the perceptions and attitudes of parents who have used BWs. Notably, 332 out of 400 (83.0%) parents reported their child using a BW, with usage typically starting between 6 and 7 months by 160 parents (48.1%). Furthermore, 151 (45.4%) of parents used walkers for all their babies, and 159 (47.8%) used a BW to promote the early waking of the child with the most common reason for using BW being for child’s fun 237 (71.3%). Most parents i.e. 146 parents (43.9%) used the walker for 1-2 hours daily, and the daily usage of BWs is seen among 186 (56%) of parents. A majority (268 parents; 80.7%) purchased the walker while making decisions on the type of BW and 209 parents (63.0%) purchased based on safety. Among the children who used walkers, a total of 48 (14.4%) experienced injuries. These injuries encompassed various types and severities. The most common type of injury was flipping over a flat surface, reported in 21 children (43.7%). This typically occurred when the walker tipped or rolled over, resulting in falls. Falling downstairs was another significant injury, affecting 20 children (41.6%). In these cases, the child gained momentum and lost control, leading to falls down the stairs. Twelve children (25%) suffered injuries from colliding with hard objects, such as furniture or walls, while using the walker. Accessing dangerous items was also a concern, with nine children (18.7%) being injured due to interactions with potentially hazardous objects such as sharp utensils, hot surfaces, or toxic substances.

**Table 3:** Perception and attitude of parents who used baby walkers

		Frequency (n=332)	Percent
Does the Child Use Baby Walker (Total =400)	Yes	332	83.0
Age of Child When Using Baby Walker	6-7 Months	160	48.1
	8 - 9 Months	118	35.5
	10 – 11 Months	44	13.2
	12-18 Months	10	3.1
How Many of Your Children Use Baby Walker	1 Child	66	19.8
	2-3 Children	79	23.7
	Most of my children	36	10.8
	All My Children	151	45.4
Reason for Using Baby Walkers	Mother can do Household Work	161	48.4
	To keep the Baby Busy	87	26.2
	Father’s or Grandparent’s Wish	14	4.2
	To Strengthen the Baby's Legs	90	27.1
	Promotes Early Walking	159	47.8
	Fun for Child	237	71.3
Daily Use of Walker	< 1 Hour a daily	95	28.6
	1-2 hours daily	146	43.9
	2-4 hours daily	64	19.3
	> 4 hours a day	27	8.1
Weekly Use of Walker	Daily	186	56.0
	Every Other Day	50	15.1
	1-2 Days per Week	19	5.7
	Most days of the week	77	23.2
Origin of Baby Walker	Purchased	268	80.7
	Transferred	23	6.9
	Borrowed	41	12.3
Decision-Making on the Type of Baby Walker	Budget	41	12.3
	Material	23	6.9
	Safety	209	63.0
	Brand	59	17.8
A child Got Injured due to Baby Walker Use	Yes	48	14.4
What Type of Injury (n=48)	Flipping over a Flat Surface	21	43.7
	Falling Downstairs	20	41.6
	Hitting a Hard Object	12	25
	Accessing Dangerous Items	9	18.7

Table 4 shows that out of a total of 400 children, 68 (17%) do not use BWs. Among the reasons for not using BWs, the most common are concerns about affecting the child's walking 29 (42.6%), and the potential for injuries 28 (41.1%). Other reasons include a lack of pediatrician's suggestion 21 (30.8%), no specific reason 13 (23.5%), the baby walked early and didn’t need a walker 8 (11.7%), the baby feeling bored 7 (10.2%), and the fear of harm to the baby's genitals 5 (7.3%).

**Table 4:** Reasons and Beliefs of Parents who don’t use a baby walker

		Frequency (n=68)	Percent
Does the child use a baby walker (total =400)	No	68	17.0
Why don’t you use a baby walker	Affect baby's walking	29	42.6
	Associated with injuries	28	41.1
	Pediatrician didn’t suggest	21	30.8
	No reason	13	23.5
	Baby walked early (didn’t need)	8	11.7
	Baby feels bored	7	10.2
	Harm to baby’s genitals	5	7.3

**Figure 1:**
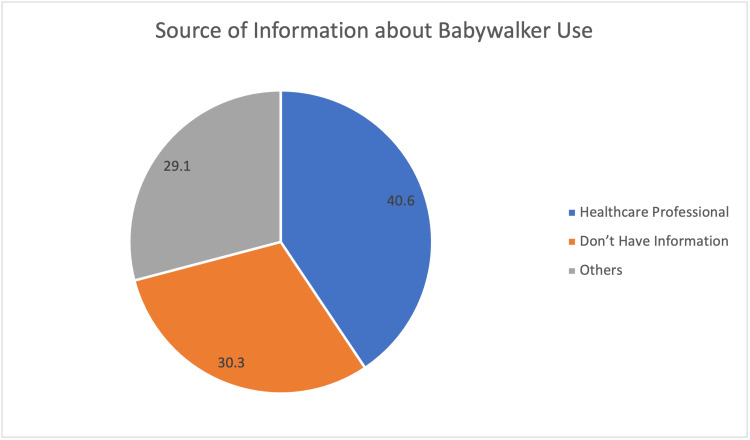
Source of information about baby walker use

Figure 1 shows the common sources for parents about BW use. A healthcare professional is the most common source for BW usage for 160 parents (40.6%).

Table 5 shows the association between parents' use of BW usage and various sociodemographic features. Notably, there is a significant relationship between mothers' age and use of BWs, with mothers 21-30 years having a higher likelihood of using BWs (p=0.042). Mother's education level showed no significant association (p=0.168). Similarly, mother's nationality, place of residence, working status, and caregiver presence at home did not significantly impact their use of BW (p>0.05). However, there was a significant association between BW use and the order of the child (p<0.001) (first child uses more walker), age of child crawling (p<0.001) (baby start crawling at 6-8 months who use a BW), and age of child beginning to walk independently (p<0.001) (baby start walking independently at 12-15 months who use a BW). 

**Table 5:** Association of parent’s usage of a baby walker with sociodemographic features

		Use of Baby Walker		Sig. Value
		Baby Walker is Not Used	Baby Walker is Used	
Mother's Age	< 20 Years	6	58	0.042
	21-30 Years	32	196	
	31-40 Years	19	51	
	41-50 Years	0	6	
	51-60 Years	0	3	
Mother's Education	< High School	1	19	0.168
	High School	9	63	
	University	58	250	
Mother's Nationality	Saudi	67	320	0.705
	Non-Saudi	1	12	
Place of Residence	Dammam	18	90	0.390
	Dhahran	5	24	
	Hasa	29	134	
	Jubail	0	12	
	Khobar	9	55	
	Qatif	7	17	
Mother's Work Status	Yes	30	137	0.664
Caregiver in Home	Yes	21	131	0.184
Any Family Support to Mother	Yes	59	270	0.285
Child’s Features				
Gender of Child	Male	28	169	0.144
	Female	40	163	
No. of Child	1	22	90	0.380
	> 1	46	242	
Order of Child	First	30	232	<0.001
	Middle	7	36	
	Last	31	64	
Age of Child Crawling	Didn't Crawl Yet	9	4	<0.001
	4-6 Months	14	95	
	6-8 Months	32	165	
	9-11 Months	9	60	
	12 Months & Above	4	8	
Age of Child Begins to Walk Independently	Didn't Independently Walk Yet	15	16	<0.001
	Before 12 Months	12	99	
	12-15 Months	36	187	
	15-18 Months	3	23	
	After 18 Months	2	7	

## Discussion

The purpose of this study was to investigate parental beliefs, attitudes, and practices related to the use of BWs. Our findings shed light on several key aspects, including the prevalence of BW usage among different age groups, the influence of education and employment status, the timing of walker usage in relation to infant developmental milestones, reasons for using BWs, safety considerations, and concerns expressed by parents who choose not to use walkers.

The high prevalence of BW usage among parents aged 21-30 years suggests that younger parents are more open to adopting new childcare practices or technologies. This finding aligns with the notion that younger parents may be more receptive to incorporating BWs into their parenting routines. Contrary to some earlier studies that linked lower maternal education to walker use, our study found that a significant proportion of mothers with a university education also utilized BWs. This indicates that higher education levels may not necessarily deter parents from using BWs, challenging previous assumptions. For example, Dogan et al. (2009) reported a negative association between lower maternal education and BW use [6]. Similarly, our study found that working mothers, comprising 41.2% of the participants, were not averse to using BWs, possibly due to the convenience they offer. This aligns with the findings of Çatakli et al. (2023), who reported that working mothers used BWs significantly more than housewife mothers [7]. These results highlight the importance of considering socioeconomic factors when examining BW usage patterns.

It is noteworthy that a significant proportion of infants in our study began crawling between 4 and 8 months, which coincides with the period when BWs are often introduced. This finding supports the notion that BWs are used to keep infants engaged and mobile during the crawling stage. Furthermore, most babies in our study walked independently between 12 and 15 months, indicating that parents might use walkers to encourage early walking. This finding contradicts the results of a previous study by Siegel et al. (1999), which reported a delayed onset of walking among children who used BWs [8]. Additionally, our study revealed that the majority of parents (83%) reported that their child had used a BW, with usage often starting as early as 6-7 months. This aligns with the perception that BWs are commonly used to facilitate mobility before infants can walk independently. Yaghini et al. (2020) also found that parents believed BWs can promote earlier walking, with an average starting age of 6.61 ± 1.46 months [9]. The high proportion of parents (45.4%) who used walkers for all of their children suggests a sense of continuity in walker usage patterns within families.

One of the most common reasons reported by parents for using BWs was to promote early walking, indicating a strong belief that walkers play a role in accelerating a child's walking development. This finding aligns with the results of Alessa et al. (2015), who reported that the most common reason for using BWs was to promote early walking [10]. Parents often seek tools and devices that can aid in their child's development, and the belief in the positive effects of walkers on early walking serves as a significant driver for their usage.

Another noteworthy finding is that a large proportion of parents (71.3%) mentioned that using a BW was fun for the child. This suggests that parents perceive walkers as a source of entertainment and engagement for their infants. Fun and entertainment are additional factors contributing to the popularity of BWs among parents, as found in the study by Laffoy et al., (1995) which showed that baby’s enjoyment and fun were the most common reasons to use BWs [11]. 

In terms of usage patterns, the majority of parents 146 (43.9%) used the walker for 1-2 hours daily, which is consistent with the previous research by Serrano et al., (1996) which shows that 46.7% of parents used BWs daily [12]. However, it is crucial to consider safety aspects when evaluating the benefits of using BWs. In our study, the majority of parents (63%) made decisions on walker usage based on safety considerations. This finding is consistent with a previous medical study by DiLillo et al., which reported that 88% of mothers were aware of the associated risks and cited safety as the most common reason for abstaining from walker usage [13]. It is important to note that while parents often choose BWs based on safety standards, this heightened perception of safety can lead to increased reliance on the walker's safety measures, potentially raising the risk of injuries.

Regarding safety concerns, in our study, a notable 14.4% of children who used walkers experienced injuries, indicating a concerning prevalence of harm. The finding is consistent with a study reported by Gaw et al., which showed 16.2% of nursery product-related injuries were most commonly associated with BWs [14]. Flipping over on a flat surface and falling downstairs emerged as the most common types of injuries, underscoring the potential for falls and subsequent injuries. Collisions with objects and accessing hazardous items were also reported, further emphasizing the diverse range of risks associated with BW usage. Similarly, the study by Barss et al. revealed a high incidence of injuries among families using BWs, with 87% reporting injuries. The documented injuries resulted in emergency room visits, hospitalizations, disabilities, and even deaths, reinforcing the severity of the risks involved. The study identified hitting objects, overturning, accessing hazardous objects, and falling downstairs as the primary causes of injuries [15]. Collectively, these findings provide compelling evidence of the significant risks and adverse outcomes associated with BW use. To address these risks, it is crucial to educate parents, caregivers, and healthcare professionals about the potential dangers associated with BWs. Emphasizing safe usage guidelines, such as avoiding stairs, ensuring a hazard-free environment, and increasing parental supervision might help reduce the incidence of injuries. 

Importantly, approximately 17% of parents in our study chose not to use BWs for variable reasons including concerns about reduced motor development, increased risk of injuries, and a preference for alternative methods of promoting infant mobility. The majority of our targeted population held the belief that the utilization of BWs could have adverse effects on infant walking development (42.6%) and were associated with a risk of injuries (41.1%), aligning with the recommendations of the AAP which advise against the use of this device due to high prevalence of accidents, including falls on staircases and head traumas [16]. This collective parental concern emphasizes the potential serious consequences of injuries during infancy, underscoring the need for proactive measures to ensure the safety and well-being of infants in their early developmental stages. 

Additionally, our study revealed that 23% of those participants who avoid using BWs avoid it for no reason. In accordance with the findings of a previous study by Bar-on, 22% of respondents held the belief that walkers did not offer any advantages for their children while others were uncertain regarding the rationale behind the contraindication of BWs [17]. On the other hand, 7.3% of our population avoid BWs so as not to harm the baby’s genitals. To the best of our knowledge, there is no substantial evidence to suggest that using BWs specifically harms the genitals. While there is no direct link to harm to the genitals, the overall safety concerns associated with BWs highlight the importance of careful consideration when choosing baby equipment. 

For parents, it is essential to weigh the perceived benefits of BWs against the potential risks. While BWs may offer convenience and entertainment, parents should be cautious and follow safety guidelines to reduce the risk of injuries. They should also explore alternative methods, such as supervised floor play and age-appropriate toys, to promote their child's motor development.

This study has some limitations that should be taken into consideration. First, the sample size of 400 mothers in the Eastern region of Saudi Arabia may not fully represent the diversity of parental beliefs and attitudes toward BWs in the entire population. Second, the data collected in this study relied on self-reported information, which introduces the possibility of recall bias and social desirability bias. Participants may not accurately remember or report their beliefs, attitudes, and practices regarding BW usage and may also provide responses that are perceived as socially desirable. Furthermore, an additional limitation of this study was that there are 29 missing data out of 400 in the domain of the mother’s age only. This limitation was likely due to some misunderstanding of the mother’s age part of the questionnaire, as some mothers wrote their child’s age instead of their own when responding to the survey.

## Conclusions

Our study provides a comprehensive understanding of parental beliefs, attitudes, and practices related to BW usage. The data reveal that while many parents use BWs to promote early walking and provide entertainment for their infants, concerns about safety and potential developmental impacts persist. Understanding these factors is crucial for policymakers, healthcare professionals, and parents in making informed decisions regarding the use of baby walkers to ensure the safety and well-being of infants. Further research could delve into the long-term developmental outcomes and safety implications of BW usage to guide evidence-based recommendations.
